# Characterization of erenumab and rimegepant on calcitonin gene-related peptide induced responses in *Xenopus Laevis *oocytes expressing the calcitonin gene-related peptide receptor and the amylin-1 receptor

**DOI:** 10.1186/s10194-022-01425-9

**Published:** 2022-05-26

**Authors:** Sanne Hage La Cour, Kiki Juhler, Lisette J. A. Kogelman, Jes Olesen, Dan Arne Klærke, David Møbjerg Kristensen, Inger Jansen-Olesen

**Affiliations:** 1 Danish Headache Center, Department of Neurology, Rigshospitalet, University of Copenhagen, Copenhagen, Denmark; 2 Department of Veterinary and Animal Sciences, Faculty of Health and Medical Sciences, University of Copenhagen, Frederiksberg C, Denmark; 3 Inserm (Institut national de la santé et de la recherche médicale), Irset - Inserm UMR 1085, Rennes, France; 4 Department of Biology, University of Copenhagen, Copenhagen, Denmark

**Keywords:** Migraine, CGRP, *Xenopus Laevis* oocytes, Amylin, Amylin receptor, RAMP1, CLR, CTR

## Abstract

**Background:**

The clinical use of calcitonin gene-related peptide receptor (CGRP-R) antagonists and monoclonal antibodies against CGRP and CGRP-R has offered new treatment possibilities for migraine patients. CGRP activates both the CGRP-R and structurally related amylin 1 receptor (AMY_1_-R). The relative effect of erenumab and the small-molecule CGRP-R antagonist, rimegepant, towards the CGRP-R and AMY-R needs to be further characterized.

**Methods:**

The effect of CGRP and two CGRP-R antagonists were examined in *Xenopus laevis* oocytes expressing human CGRP-R, human AMY_1_-R and their subunits.

**Results:**

CGRP administered to receptor expressing oocytes induced a concentration-dependent increase in current with the order of potency CGRP-R> > AMY_1_-R > calcitonin receptor (CTR). There was no effect on single components of the CGRP-R; calcitonin receptor-like receptor and receptor activity-modifying protein 1. Amylin was only effective on AMY_1_-R and CTR. Inhibition potencies (pIC_50_ values) for erenumab on CGRP induced currents were 10.86 and 9.35 for CGRP-R and AMY_1_-R, respectively. Rimegepant inhibited CGRP induced currents with pIC_50_ values of 11.30 and 9.91 for CGRP-R and AMY_1_-R, respectively.

**Conclusion:**

Our results demonstrate that erenumab and rimegepant are potent antagonists of CGRP-R and AMY_1_-R with 32- and 25-times preference for the CGRP-R over the AMY_1_-R, respectively. It is discussed if this difference in affinity between the two receptors is the likely reason why constipation is a common and serious adverse effect during CGRP-R antagonism but less so with CGRP binding antibodies.

**Supplementary Information:**

The online version contains supplementary material available at 10.1186/s10194-022-01425-9.

## Background

The sensory peptide calcitonin gene-related peptide (CGRP) has been shown to be an important molecule in migraine pathophysiology [1]. Its importance has been further established by the development of CGRP receptor (CGRP-R) antagonists and monoclonal antibodies against CGRP and CGRP-R offering a new treatment avenue for migraine [2, 3].

CGRP and CGRP-R are widely distributed in the human organism with high abundancy in the CNS [4, 5], gastrointestinal tract and cardiovascular system [6]. In addition to its effect on CGRP-R, CGRP also potently activates the amylin 1 receptor (AMY_1_-R) [7]. The CGRP-R is formed by receptor activity-modifying protein 1 (RAMP1) and the calcitonin receptor-like receptor (CLR), while the AMY_1_-R consists of RAMP1 and the calcitonin receptor (CTR) [8, 9]. In the trigeminovascular and gastrointestinal systems CGRP-R and AMY_1_-R have been identified [10, 11].

Although most of the research concerning the specificity of monoclonal antibodies and CGRP-R antagonists for the CGRP-R, it must be taken into account that CGRP-R antagonists also induce an effect via AMY_1_-R as described for CGRP. Moreover, the relative potency of the monoclonal antibody erenumab targeting CGRP-R and AMY_1_-R needs to be futher characterized [3]. Early studies used the MCF-7 cell line, suggested to contain CTR, and amylin receptors [12], in combination with assays of cAMP levels and radioactive binding to characterize the effect of erenumab [13]. However, it was unclear from these studies if; (*i*) CGRP-R is expressed by the MCF-7 cell line; (*ii*) if CGRP binds AMY_1_-R in the MCF-7 cell line; and whether erenumab can block CGRP activation of AMY_1_-R in the MCF-7 cell line [12, 13]. After finishing the experimental part of this study, two studies were published that elegantly showed the binding of erenumab and rimegepant to CGRP-R and AMY_1_-R [14, 15].

We here investigate the effect of CGRP and amylin on *Xenopus laevis* oocytes expressing CGRP-R, AMY_1_-R and their subunits. Furthermore, the CGRP-R antibody erenumab and the antagonist rimegepant were studied on CGRP induced responses in oocytes expressing CGRP-R and AMY_1_-R. We further discuss the clinical relevance for our findings.

## Materials and methods

### Analysis of transcriptomic expression of CLR, CTR, RAMP1, RAMP2 and RAMP3 in MCF-7 cells

Publicly available RNA-sequencing data of MCF-7 cells was downloaded from the Gene Expression Omnibus database (GSE130852). In short, the data consisted of eight MCF-7 samples that were sequenced using the Illumina NextSeq 500 sequencer in two separate batches and were aligned to the human reference genome (hg19) using STAR. Feature counts were produced using HTSeq, as used in the present study. Raw feature counts were converted into transcripts per million (TPM) to correct for gene length and sequencing depth.

### Molecular biology and cloning

cDNA coding for the human CTR transcript variant 2 (CT_(a)_ subtype) (NM_001742.4), human CLR (NM_005795.5) and human RAMP1 (NM_005855.3) were purchased from GenScript (Leiden, Netherlands) in the pcDNA3.1(+) plasmid. A kozak element was added in the beginning of the different cDNAs. The cDNA encoding the three different receptor subtypes were cleaved by BamHI and NotI and ligated into the pXOOM vector, which have been optimized to the *Xenopus laevis* oocytes expression system [16]. DNA was purified using Plasmid DNA purification NucleoBond Xtra Midi-kit (Macherey-Nagel, Tilst, Denmark). DNA was sequenced to confirm correct insertion the (data not shown) (GATC Eurofins, Ebersberg, Germany).

### In vitro transcription

Thirty microgram extracted plasmids were linearized down-stream the poly(A) segment using the *XhoI* restriction enzyme (New England Biolabs, Ipswich, MA, USA) at 37 °*C* overnight. The linearized plasmids were purified using the High Pure PCR purification kit (Roche, Hvidovre, Denmark) according to manufactures protocol. The plasmid DNA was in vitro transcribed to messenger RNA by synthetization from the T7 RNA polymerase promoter using the mMESSAGE mMASHINE kit (Invitrogen™, Waltham, MA, USA) according to manufactures protocol. Messenger RNA was purified using the MEGAclear kit (Invitrogen™, Waltham, MA, USA). Transcribed RNA integrity was assessed by agarose gel electrophoresis and the concentrations were measured by NanoDrop^TM^ 2000c (Thermo Fischer Scientific, Lillerød, Denmark).

### Two-electrode voltage clamp

Stage V-VI defolliculated *Xenopus laevis* oocytes (EcoCyte Bioscience, Dortmund, Germany) were kept in Kulori medium (90 mM NaCl, 4 mM KCl, 1 mM MgCl_2_, 1 mM CaCl_2_, and 5 mM Hepes, pH 7.4) at 19 °*C* upon delivery. Oocytes were micro-injected with 50 nl mRNA containing either 5 ng RAMP1 together with 5 ng CRL to form the CGRP-R og 5 ng RAMP1 with 5 ng of CTR to form the AMY_1_-R. To insure that CRL/CTR is recruited to the membrane the stoichiometric ratio of RAMP1:CLR/CTR is 3:1. Oocytes were also injected with 5 ng RAMP1, 5 ng CTR, or 5 ng CLR. Injected oocytes were kept in Kulori medium at 19 °C and currents were measured using conventional two-electrode voltage clamp (TEVC). Recording electrodes were pulled from glass capillaries (TW122.3, World Precision Instruments, Hitchin, Hertfordshire, SG4 0 T, UK) and had a resistance of 0.5-1.5 MΩ. For measurements, oocytes were placed in a 200 μl chamber and continuously exposed to Kulori medium at a flowrate of 1 ml min^− 1^ and with a temperature of 19 °*C*. The electrodes were connected to an Oocyte Clamp Amplifier OC-725 B (Warner Instruments Corp., Holliston, MA 01746) and data were sampled at 2 kHz through an Axon Digidata 1440A digitizer using the pClamp 10.4 acquisition software. The current change induced by human *α*–CGRP or human amylin were first investigated in oocytes expressing AMY_1_-R or CGRP-R by a voltage ramp from − 80 to + 40 mV (10 mV increments of 350 ms) from a holding potential of − 60 mV. Activation of human *α*–CGRP and human amylin were investigated in oocytes injected with AMY_1_-R, CGRP-R, CTR, RAMP1 and CLR by application of concentrations ranging from 0.01 μM to 10 μM for 30 seconds at a holding potential of − 70 mV. These responses were compared to the effect observed in non-injected oocytes referred to as controls. Inhibition by erenumab and rimegepant were investigated by a 5 min pre-incubation period with erenumab or rimegepant in concentrations ranging from 1 pM to 1 μM for oocytes expressing CGRP-R and 0.1 nM to 1 μM for oocytes expressing AMY_1_-R. After the pre-incubation, 1 μM and 3 μM human *α*-CGRP were applied to CGRP-R and AMY_1_-R expressing oocytes, respectively for 30 sec at a holding potential of − 70 mV. The concentrations of human *α*-CGRP given above, were the ones that caused the maximum response at each of the two receptors.

### Experimental protocol

Currents were measured 20-48 hours after injections. Human *α*–CGRP and human amylin were administered in different concentrations to oocytes expressing the different receptors and their subunits alone. Due to receptor desensitization it was not possible to repeat measurements on individual oocytes, thus only one dose could be tested per oocyte. Effort was made to run experiments with several different concentrations of peptides on each batch of oocytes. In the blocking experiments the oocytes were pre-incubated with the different antagonists 2-5 minutes before activation. In the dose-response experiments non-responding as well as low-responding (< 10 nA) oocytes were excluded from the dataset. All the figures are based on several batches of oocytes. Due to variation in the expression level between different batches of oocytes, the results of the blocking experiments are given in % of the response to CGRP (1 μM at the CGRP-R and 3 μM at the AMY_1_-R) when given alone.

### Compounds

Human amylin, human *α*-CGRP and Rimegepant were obtained from Tocris (Abingdon, United Kingdom). All substances were dissolved in distilled water and stored as aliquots at − 20 °*C*. Erenumab (AMG334) was obtained from Amgen (Thousand Oaks, CA, USA), it was delivered in a stock solution and stored at 4 °*C*. At the day of use the stock of compounds to be used were further diluted in kulori.

### Statistical analysis

Current amplitudes were analyzed with pClamp 10.2 software (Molecular Devices San Jose, CA, USA). GraphPad Prism 8.0.0 (GraphPad Prism Software, San Diego, CA, USA) was used for statistical analysis. For curve fittings, a non-linear regression curve using least square non-lin fit of the c-r relationship of log(agonist) vs. response (three parameters) were performed. Specific information of the curve fittings are given in the legend to the figures. Concentration-response curves of CGRP and amylin on the different receptors and subtypes were compared to their effect on non-injected control oocytes by ordinary one-way ANOVA followed by Dunnett’s test for multiple comparisons. The data are presented as mean with standard error of mean (±SEM) and differences between groups were considered significant when *p* < 0.05.

## Results

### MCF-7 cells express AMY_1_-R and AMY_3_-R but not the CGRP-R

**Fig. 1:**
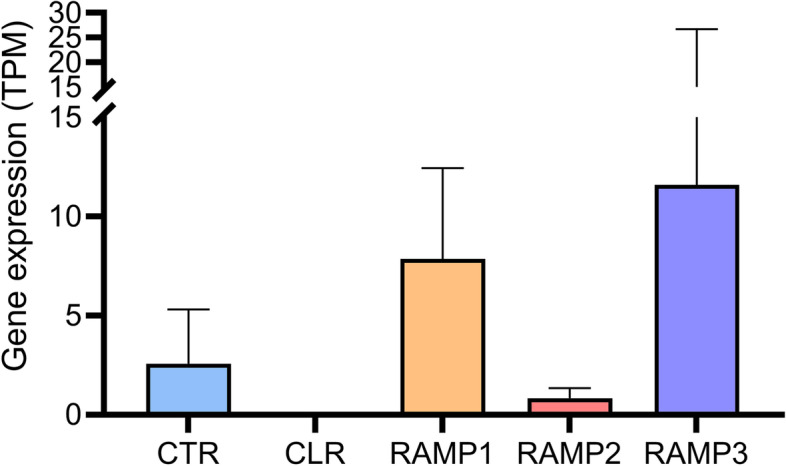
Based on publicly available RNAseq data from MCF-7 cells, we investigated the expression of the different receptor subunits; CTR, CLR, RAMP1, RAMP2 and RAMP3. Based on this we observed the MCF-7 cell line most probably expresses functional CTR, AMY_1_-R and AMY_3_-R

Using publicly available RNAseq data [17], we investigated the expression of the genes CLR (i.e. CALCRL), CTR (i.e. CALCR), RAMP1, RAMP2 and RAMP3. It was found that CLR was not expressed in the MCF-7 cells. However, the MCF-7 cell line does express CTR, RAMP1 and RAMP3 (Fig. 1). Thus, this cell line has the potential to have functional CTR, AMY_1_-R and AMY_3_-R.

### Human CGRP and amylin induce an inward chlorine current in *Xenopus laevis* oocytes expressing CGRP-R or AMY_1_-R

**Fig. 2:**
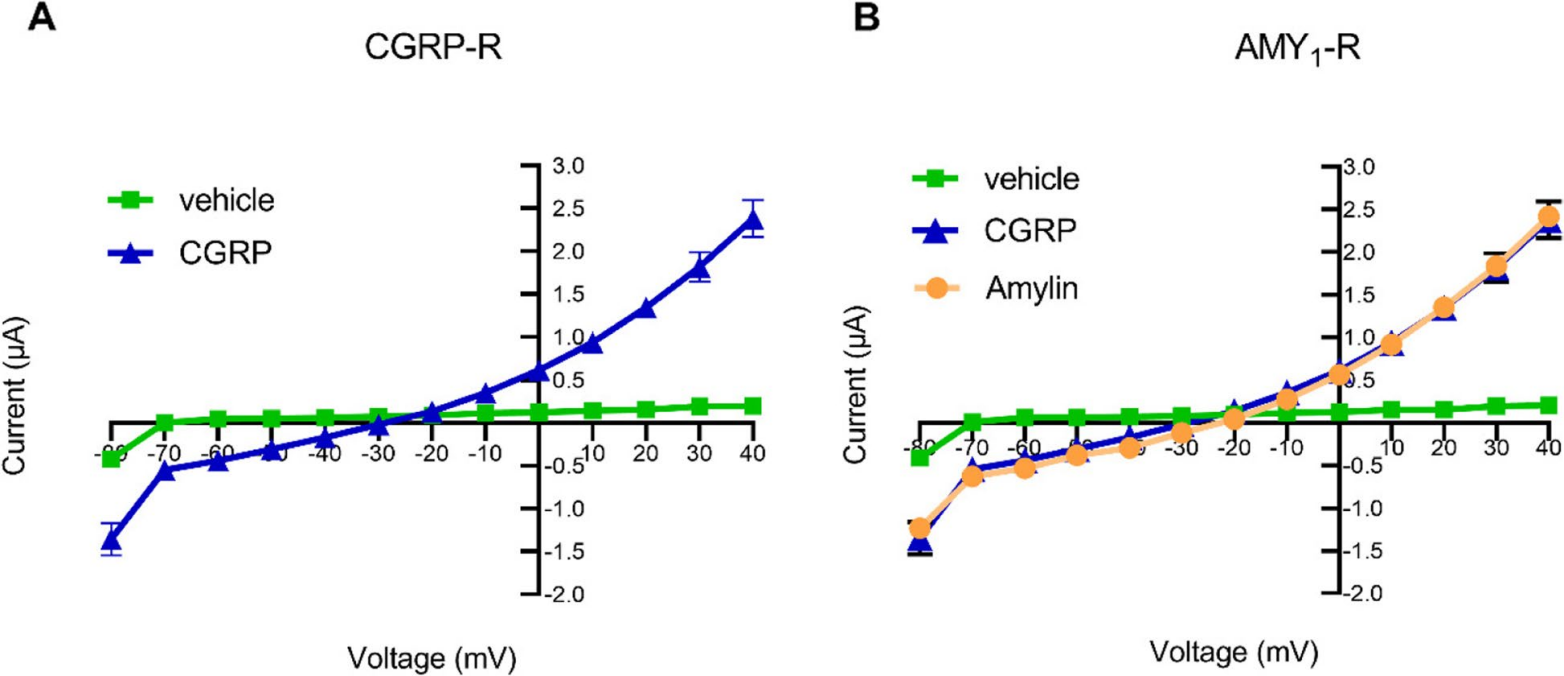
Current voltage relationships for oocytes expressing **A.** CGRP-R in the presence of 1 μM CGRP and vehicle and **B**. AMY_1_-R in the presence of 1 μM CGRP, 3 μM Amylin and vehicle. Currents were elicited by a voltage ramp from − 80 to 40 mV in 10 mV increments (350 ms). Data is presented as mean with standard error of mean (±SEM), *n* = 4

We compared the change in current induced by human CGRP and human amylin in *Xenopus laevis* oocytes expressing AMY_1_-R or CGRP-R. Currents were recorded by a voltage ramp from − 80 to + 40 mV (10 mV increments of 350 ms) from a holding potential of − 60 mV. The shown currents at each voltage were measured at the peak of the current induced by agonist applied for 30 sec. Under these conditions, human CGRP (1 μM) added to CGRP-R expressing oocytes induced a rapid inward current (reversal potential − 20 to − 30 mV) (Fig. 2A). When human CGRP (1 μM) or human amylin (3 μM) were administered to AMY_1_-R expressing oocytes, currents were observed for both peptides (Fig. 2B). The currents observed from activation of the AMY_1_-R and CGRP-R were consistent with activation of an endogenous chloride current as compared to Kulori (vehicle) only (Fig. 2).

### The effect of human α-CGRP on *Xenopus laevis* oocytes expressing CGRP-R, AMY_1_-R or their subunits

**Fig. 3:**
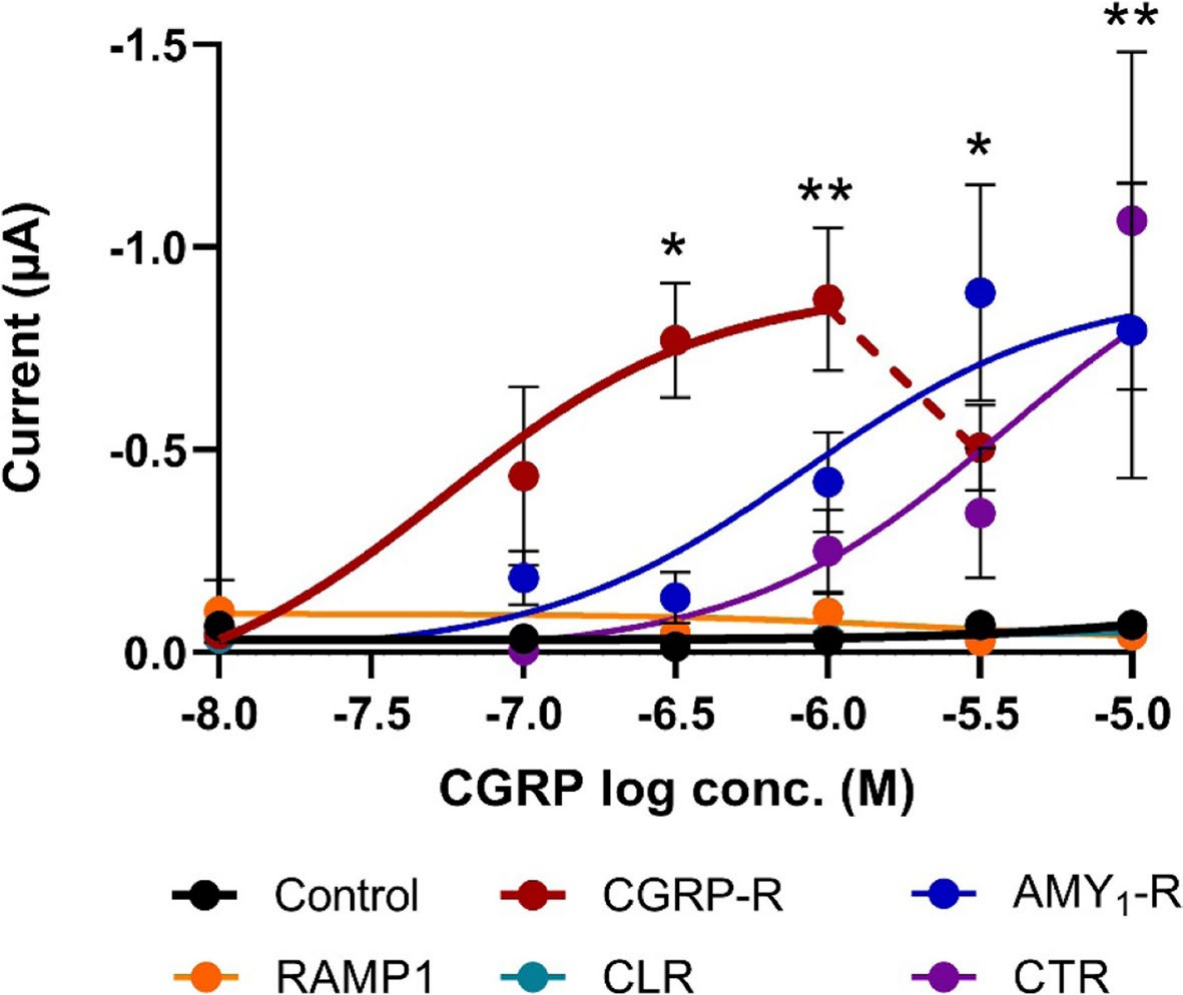
Application of CGRP to *Xenopus laevis* oocytes expressing CGRP-R, AMY_1_-R and CTR caused a concentration-dependent increase in current at a holding potential of − 70 mV with CGRP being most potent on CGRP-R before the AMY_1_-R and CTR. CGRP had no effect on oocytes expressing RAMP1, CLR or non-injected oocytes (control). Curve fittings by non-linear regression curves using least square non-lin fit of the c-r relationship of log(agonist) vs. response (three parameters) were performed. The top and the bottom of the curves were constrained to the maximum response of CGRP and zero, respectively for CGRP-R, AMY_1_-R and CTR. Furthermore, the response to 3 μM of CGRP at the CGRP-R was not included in the calculation of the non-linear regression curve (indicated by dashed line) Statistical evaluation was performed by ordinary One-way ANOVA followed by Dunnet’s multiple comparisons test on the response to the different concentrations of CGRP at each receptor as compared to the 1 μM CGRP response in non-injected control oocytes * *p* < 0.05; **p < 0.01. The number of experiments performed at each data point is between 2 and 15. Each curve-fitting relies on experiments performed on 13 non-injected oocytes, and 36, 30, 20, 14 and 27 oocytes expressing CGRP-R, AMY_1_-R, RAMP1, CLR and CTR, respectively

CGRP caused a significant concentration-dependent inward current when administered to *Xenopus laevis* oocytes expressing CGRP-R, AMY_1_-R or CTR at a holding potential of − 70 mV (Fig. 3). The order of potency was CGRP-R (pEC_50_ = 7.26) > > AMY_1_-R (pEC_50_ = 6.08) > CTR (pEC_50_ = 5.42). The maximum currents observed in the presence of CGRP was − 0.87 + 0.17 μA (1 μM) for the CGRP-R, − 0.89 + 0.27 μA (3 μM) for the AMY_1_-R and − 1.07 + 0.42 μA (10 μM) for CTR expressing *Xenopus laevis* oocytes. At the highest concentrations of CGRP used at the CGRP-R (3 μM) and the AMY_1_-R (10 μM), the measured responses declined (biphasic response). At the CTR it was not possible to investigate the effect of CGRP at higher concentrations than 10 μM, which induced the maximum response.

No significant response to CGRP (0.01 to 10 μM) was observed in RAMP1 and CLR expressing *Xenopus laevis* oocytes. In these experiments, the maximum current observed was < − 0.03 μA and − 0.04 μA at 10 μM CGRP for the RAMP1 and CLR expressing oocytes, respectively (Fig. 3).

### The effect of human amylin on *Xenopus laevis* oocytes expressing CGRP-R, AMY_1_-R or their subunits

**Fig. 4:**
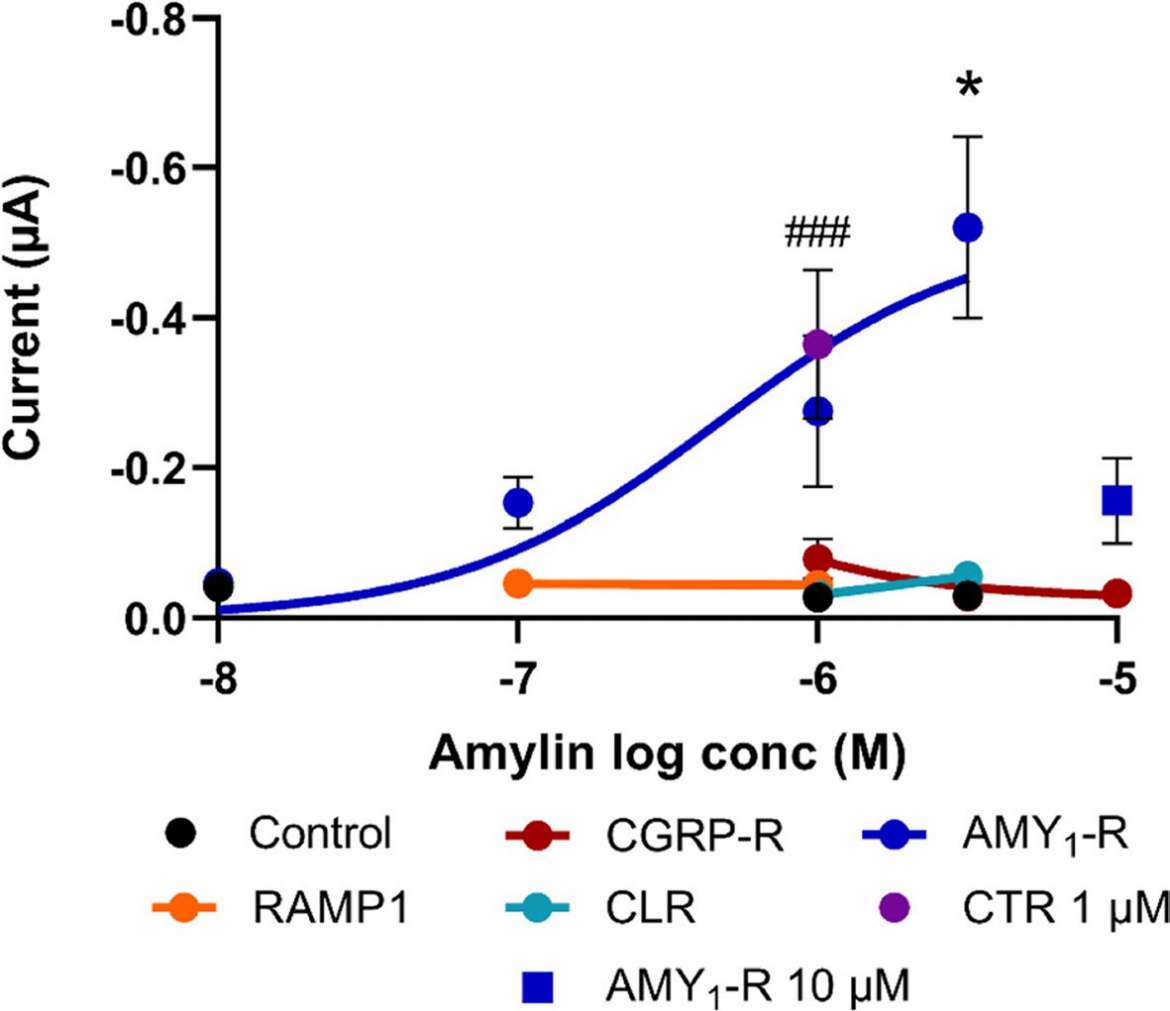
Application of Amylin to *Xenopus laevis* oocytes expressing AMY_1_-R caused concentration-dependent increase in current at a holding potential of − 70 mV. In CTR expressing oocytes a significant response was obtained at 1 μM amylin. Amylin had no effect on oocytes expressing CGRP-R, RAMP1, CLR or non-injected oocytes (control). Curves fittings by non-linear regression curves using least square non-lin fit of the c-r relationship of log(agonist) vs. response (three parameters) were performed. The top and the bottom of the AMY_1_-R curve was constrained to the maximum response of amylin and zero, respectively. Furthermore, the response to 10 μM of amylin at the AMY_1_-R was not included in the calculation of the non-linear regression curve. Statistical evaluation was performed by ordinary One-way ANOVA followed by Dunnet’s multiple comparisons test on the response to the different concentrations of Amylin at one receptor at the time as compared to the 1 μM amylin response in non-injected control oocytes* *p* < 0.05; **p < 0.01. Mann Whitney test was used for the comparison of Amylin 1 μM on CTR, as compared to its response in non-injected control oocytes ### *p* = 0.0010. The number of experiments performed at each data point is between 2 and 26. Each curve-fitting relies on experiments performed on 13 non-injected oocytes, and 13, 47, 7 and 7 oocytes expressing CGRP-R, AMY_1_-R, RAMP1 and CLR, respectively. The one point for CTR consists of experiments performed on 5 oocytes

Human amylin (0.01 to 10 μM) caused a concentration-dependent current change in AMY_1_-R expressing *Xenopus laevis* oocytes with a pEC_50_ value of 6.33. The responses to amylin 1 μM and 3 μM were significantly different compared to the amylin response observed in non-injected oocytes. Addition of 10 μM amylin to AMY_1_-R showed a much lower response than at 1 and 3 μM indicating a non-specific response at this concentration (Fig. 4). Moreover, 1 μM amylin also induced a significant change in current when applied to CTR-expressing oocytes. Significant currents were not observed when amylin was added to oocytes expressing CGRP-R, RAMP1, or CLR (Fig. 4).

## Blocking experiments

### The effect of erenumab on CGRP-induced current changes in oocytes expressing CGRP-R or AMY_1_-R

**Fig. 5:**
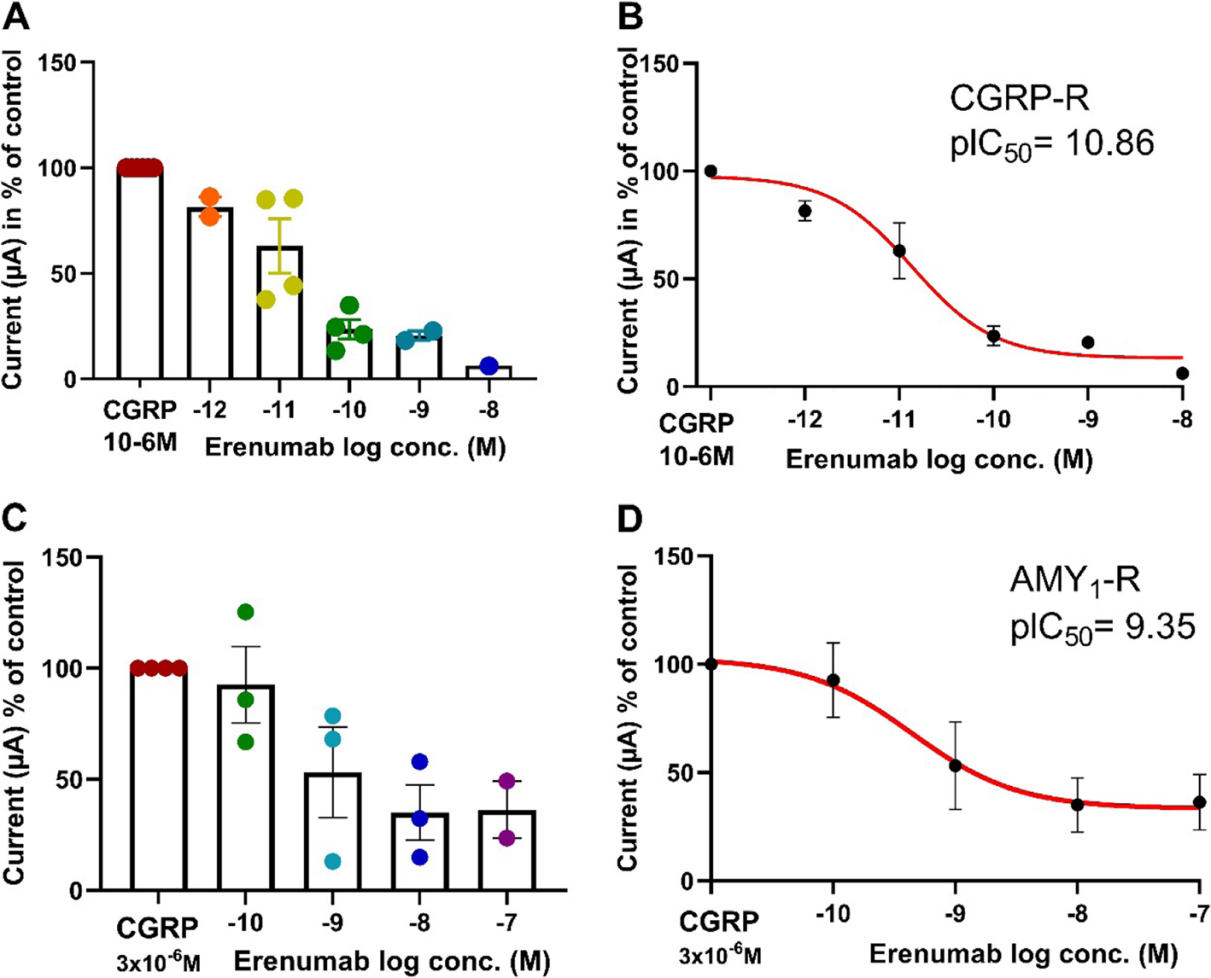
The experiments show the erenumab inhibition of CGRP induced currents in *Xenopus Laevis* oocytes expressing CGRP-R (**A** & **B**) and AMY_1_-R (**C** & **D**). In **A-D**, the currents induced by CGRP at each concentration of erenumab is given in % of the current obtained for CGRP without antagonist in 4-6 batches of oocytes. At a given concentration of erenumab in **A** & **C** each point represents the mean of 2-4 identical experiments in one batch of oocytes. In **B** & **D** the least square non-lin fit of the c-r relationship of log[erenumab] vs. response (three parameters) is shown for responses performed on CGRP-R (**A**) and AMY_1_-R (**C**). The 23 data points shown in Fig. **A** are obtained from experiments performed on 48 oocytes from six different batches. The same 23 data points are used for the curve fitting in **B**. The 15 data points shown in **C** are obtained from experiments performed on 37 oocytes from 4 different batches. The same 15 data points are used for the curve fitting in **D**

For blockade experiments, we used the concentrations of CGRP that produced the maximum change in currents on the two receptors in the above-described concentration-response curves (Figs. 3 and 4). The mean change in current after stimulation with CGRP at the CGRP-R was − 1.47 + 0.39 μA (*n* = mean values of 6 batches) and − 0.60 + 0.13 μA (*n* = mean values of 4 batches) for the AMY_1_-R. During the 5 minutes pre-incubation with erenumab there was no change in currents for the two receptors. After pre-incubation the oocytes were stimulated with 1 and 3 μM CGRP for CGRP-R (Fig. 5A and B) and AMY_1_-R (Fig. 5C and D), respectively. The CGRP responses were significantly and potently antagonized by erenumab at both receptors with pIC_50_ values of 10.86 for the CGRP-R (Fig. 5B) and 9.35 for the AMY_1_-R (Fig. 5D). Calculating the potency ratio between the two receptors, erenumab was found to be 32 times more potent for the CGRP-R as compared to the AMY_1_-R. The lowest point of the c-r curve for erenumab was 13.6% and 33.4% of the control (1 μM CGRP alone) at the CGRP-R and AMY_1_-R respectively.

### The effect of rimegepant on CGRP-induced current changes in oocytes expressing CGRP-R as compared to AMY_1_-R

**Fig. 6:**
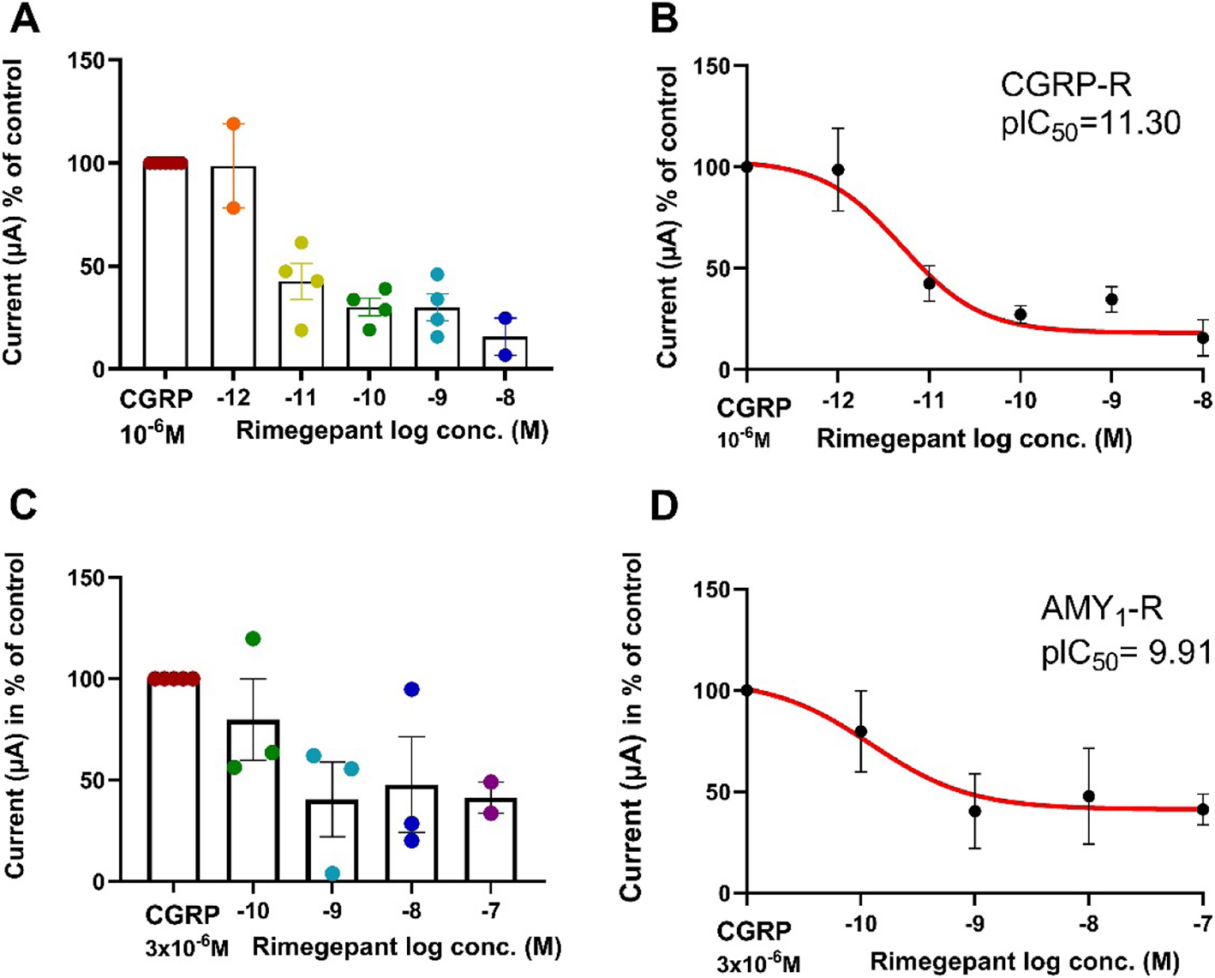
The experiments show the rimegepant inhibition of CGRP induced currents in *Xenopus Laevis* oocytes expressing CGRP-R (**A** & **B**) and AMY_1_-R (**C** & **D**). In **A-D**, the current change to CGRP at each concentration of rimegepant is given in % of the current obtained for CGRP when given alone in 5-7 batches of oocytes. At a given concentration of rimegepant in **A** & **C** each point represents the mean of 2-4 identical experiments in one batch of oocytes. In **B** & **D** the least square non-lin fit of the c-r relationship of log[rimegepant] vs. response (three parameters) is shown for responses performed on CGRP-R (**A**) and AMY_1_-R (**C**). The 23 data points shown in Fig. **A** are obtained from experiments performed on 61 oocytes from seven different batches. The same 23 data points are used for the curve fitting in **B**. The 16 data points shown in **C** are obtained from experiments performed on 36 oocytes from 5 different batches. The same 16 data points are used for the curve fitting in **D**

We next investigated the effect of rimegepant on CGRP-induced current changes in *Xenopus laevis* oocytes expressing CGRP-R and AMY_1_-R. The mean change in current when CGRP was given alone to CGRP-R was − 1.36 + 0.40 μA (*n* = mean values of 6 batches) and − 1.11 + 0.21, (*n* = mean values of 5 batches) in experiments on AMY_1_-R. The oocytes were pre-incubated with rimegepant for 5 minutes. No change in current was observed during the pre-incubation period. Subsequently the oocytes were stimulated with 1 or 3 μM CGRP depending on the receptor studied as described above. The CGRP response was antagonized by rimegepant at the CGRP-R with a pIC_50_ of 11.30 and on AMY_1_-R with a pIC_50_ of 9.91 (Fig. 6). The potency ratio of rimegepant between the two receptors was 25 times with a higher potency towards the CGRP-R than the AMY_1_-R. The lowest point of the c-r curve for rimegepant was 18.6% and 41.3% of the control (CGRP alone) at the CGRP-R and AMY_1_-R respectively.

## Discussion

In the present study, we investigated the affinity of novel CGRP receptor antagonists for the CGRP-R and AMY_1_-R. *Xenopus Laevis *oocytes injected with mRNA encoding receptors or specific receptor combinations were analyzed with two-electrode voltage clamp, and showed minimal background activity caused by endogeneous proteins. The main results of the present study are fourfold (*i*) CGRP-R is not expressed by the MCF-7 cell line; (*ii*) CGRP binds to AMY_1_-R; (*iii*) erenumab and rimegepant inhibits CGRP activation on AMY_1_-R; and (*iv*) erenumab and rimegepant have a 32- and 25-times stronger affinity for CGRP-R over AMY_1_-R, respectively.

### Concentration-response relationship

To find the most optimal concentration of agonist for these experiments, we first performed a concentration-response curve of CGRP and amylin on the different receptors and their subunits. Stimulation with CGRP induced a concentration-response relationship in oocytes expressing CGRP-R, AMY_1_-R and CTR with the order of potency as mentioned (Fig. 3). We did not record a signal for CGRP in oocytes expressing CLR or RAMP1. Previous studies have described *Xenopus Laevis* oocytes to have an endogenous CLR receptor. However, in that study RNA were injected into the oocytes with cRNA encoding the cystic fibrosis transmembrane regulator (CFTR) that enhance cAMP-mediated responses [9]. Amylin only induced a response in AMY_1_-R and CTR expressing oocytes (Fig. 4). Of interest is the ~ 500 times higher concentration of CGRP required for a significant response compared to the effect of CGRP on isolated cerebral arteries [18]. The vasodilation of CGRP is mediated via the activation of adenylyl cyclase leading to an increased formation of cAMP [19]. In the present study, the endpoint was an inward chloride current measured by electrodes inserted into the oocyte membrane which is a more indirect measure for CGRP-induced responses than changes in cAMP levels. In COS-7 cells transfected with CGRP-R and AMY_1_-R the pEC_50_ values for CGRP on cAMP formation after stimulation of the two receptors were similar to the pEC_50_ value found in isolated human cerebral arteries [18, 20]. Thus, we believe that the indirect method of measurement is the reason for the lower pEC_50_ values to CGRP obtained in this study as compared to those found by measuring changes in cAMP levels in COS-7 cells and in human cerebral arteries [18, 20]. This is supported by a study showing differences in the potency of CGRP in COS-7 cells transfected with CGRP-R and AMY_1_-R dependent on the different signaling pathways measured [21]. In oocytes expressing CLR and RAMP1 together with CFTR that enhance cAMP mediated responses, CGRP was ~ 6 times more potent than in the present study [9].

.At the highest concentrations used of CGRP at the CGRP-R and the AMY_1_-R and of amylin at the AMY_1_-R, the response declined. This biphasic allosteric effect is a wellknown receptor phenomenon occurring after administration of high agonist concentrations in vitro. The exact mechanisms of this phenomenon in the present study is unknown. A direct toxic effect on the oocyte can be excluded as this would enhance the current instead of the decrease in current observed.

### Erenumab

Erenumab was designed to bind to an extracellular high-affinity binding region of CGRP-R that includes both RAMP1 and CLR, as this unique combination in the epitope would likely provide selectivity over both the adrenomedullin-R (AM-R) and AMY_1_-R [22]. Its high affinity to the CGRP-R was confirmed in two studies using SK-N-MC cells endogenously expressing human CGRP-R (Table S1). Here we show a higher affinity of erenumab to the CGRP-R (Fig. 5, Table S1). Using the MCF-7 cell line there was no antagonistic effect of erenumab on calcitonin-induced responses [13]. Calcitonin affects four different receptors, namely AMY_1_-, AMY_2_- AMY_3_ receptors and CTR. Among these CGRP mainly shows affinity to the AMY_1_-R because RAMP1 is part of this receptor [23]. We investigated publicly available RNAseq data for MCF-7 cells [17], and found that they most likely express CTR, AMY_1_-R and AMY_3_-R, but not AMY_2_-R and CGRP-R (Fig. 1). In addition, cAMP formation in the MCF-7 cells was stimulated by calcitonin that binds to CTR and not to the two components of the CGRP-R, CLR and RAMP1. This could explain why erenumab was ineffective as an antagonist in these experiments [13]. We here show that erenumab inhibits the CGRP induced response in AMY_1_-R injected *Xenopus laevis* oocytes. This is confirmed in a recent paper where erenumab was shown to inhibit binding of CGRP to the AMY_1_-R using flow cytometry in AMY_1_-R overexpressing HEK293S cells [14](Table S1). The antagonistic effect of erenumab on CGRP induced responses was 32 times larger in CGRP-R expressing oocytes than in AMY_1_-R (Table S1). In comparison, the ratio was 18 in the HEK293S cells mentioned above [14] (Table S1). It should however, be emphazised that comparisons between data in different cells with different output and agonist concentrations has limitations. We found CGRP-induced responses in oocytes injected with AMY_1_-R not to be blocked to the same extent as observed in oocytes injected with CGRP-R. This, observation was not seen in experiments on HEK293S cells transiently transfected with AMY_1_-R or CGRP-R where the maximum binding of erenumab is 93% and 98%, respectively [14].

### Rimegepant

Rimegepant is a small molecule CGRP-R antagonist currently used in the acute and preventive treatment of migraine. It binds to a hydrophobic pocket of the CGRP receptors formed by CLR and RAMP1. More specifically, Rimegapant binds to recidues T122^CLR,^ W74^RAMP1^, W84^RAMP1^, M42^CRL^, and A70^RAMP1^ [3, 24]. We found rimegepant to be 25 times more potent on CGRP-R compared to AMY_1_-R (see Table S2). In a recent paper, a 60 times higher concentration of rimegepant, than in the present study, was required to inhibit CGRP induced increase in cAMP levels in Cos7 cells (Table S2) [15]. However, still the potency ratio between the two receptors was close to that shown in our study (Table S2). As observed in the experiments with erenumab, the CGRP induced response in oocytes injected with AMY_1_-R were not blocked by rimegepant to the same extent as observed in oocytes injected with CGRP-R. In a previous study performed on Cos7 cells transfected with AMY_1_-R or CGRP-R rimegepant completely blocked CGRP-induced increase in cAMP accumulation at both receptors [15]. As neither erenumab nor rimegepant blocked the CGRP induced response completely at the AMY_1_-R (CTR + RAMP1), we speculate that although the stoichiometry for the expressed RAMP1 in relation to CTR is 3/1 to ensure that all CTR receptors are associated with RAMP1, we can not exclude a minor number of free CTR upon which CGRP can act. As erenumab and rimegepant bind to CLR and RAMP1 this effect will not be inhibited by the two antagonists.

### Clinical relevance of the present findings

Rimegepant was recently registered for the use in prophylactic and acute migraine treatment. Erenumab was registered in 2019 and is in widespread use. In clinical studies, administration of 70 mg Erenumab (s.c.) resulted in a mean C_max_ of 41.8 nM (6.1 μg/mL) [25]. Oral administration of 75 mg Rimegepant resulted in a mean C_max_ of 1.3 μM (722 ng/mL) [26]. In another study, a plasma concentration of 1.7 nM (255 mg/mL) Erenumab (70 mg s.c.) caused 50% inhibition of capsaicin-induced increase in dermal blood flow. To obtain a 99% inhibition, a plasma concentration of 7.7 nM (1134 mg/mL) Erenumab was required [27]. So far, similar studies have not been shown for Rimegepant. It is known that 96% of the Rimegepant concentration in blood is bound to plasma proteins. Thus the free concentration of Rimegepant at a C_max_ of 1.3 μM is 54 nM [25]. Taken together, the sensitivity of Erenumab and Rimegepant on CGRP evoked responses at CGRP-R and AMY_1_-R obtained in this study, far exceeds the concentrations required for effective treatment of migraine and inhibition of capsaicin-induced increase of dermal blood flow due to CGRP release. This might reflect limitations of the method used that permits an over-expression of CGRP- and AMY_1_-receptor components [15].

Constipation is a serious clinical problem that occurs in up to 65% of patients treated with the CGRP-R antibody Erenumab [28–31]. In order to direct treatment strategy with CGRP blockers and to understand the underlying mechanisms there is a need for detailed studies on the mechanisms behind this side effect. Constipation is less frequent in patients treated with the CGRP antibodies fremanezumab and galcanezumab [29]. Below we present a hypothesis where we speculate on a possible explanation for the erenumab induced constipation in relation to the lesser frequency of this side effect after treatment with the two CGRP antibodies. CGRP is involved in a peristaltic reflex to increase gastro-intestinal (GI) motility [32, 33]. Amylin inhibits gastric emptying via amylin receptors in area postrema and causes dilation of ileum [34–36]. The receptor pharmacology for amylin in combination with expressions studies of its receptors suggest the effects to be mediated via AMY_1_-R and/or AMY_3_-R [36, 37].

**Fig. 7:**
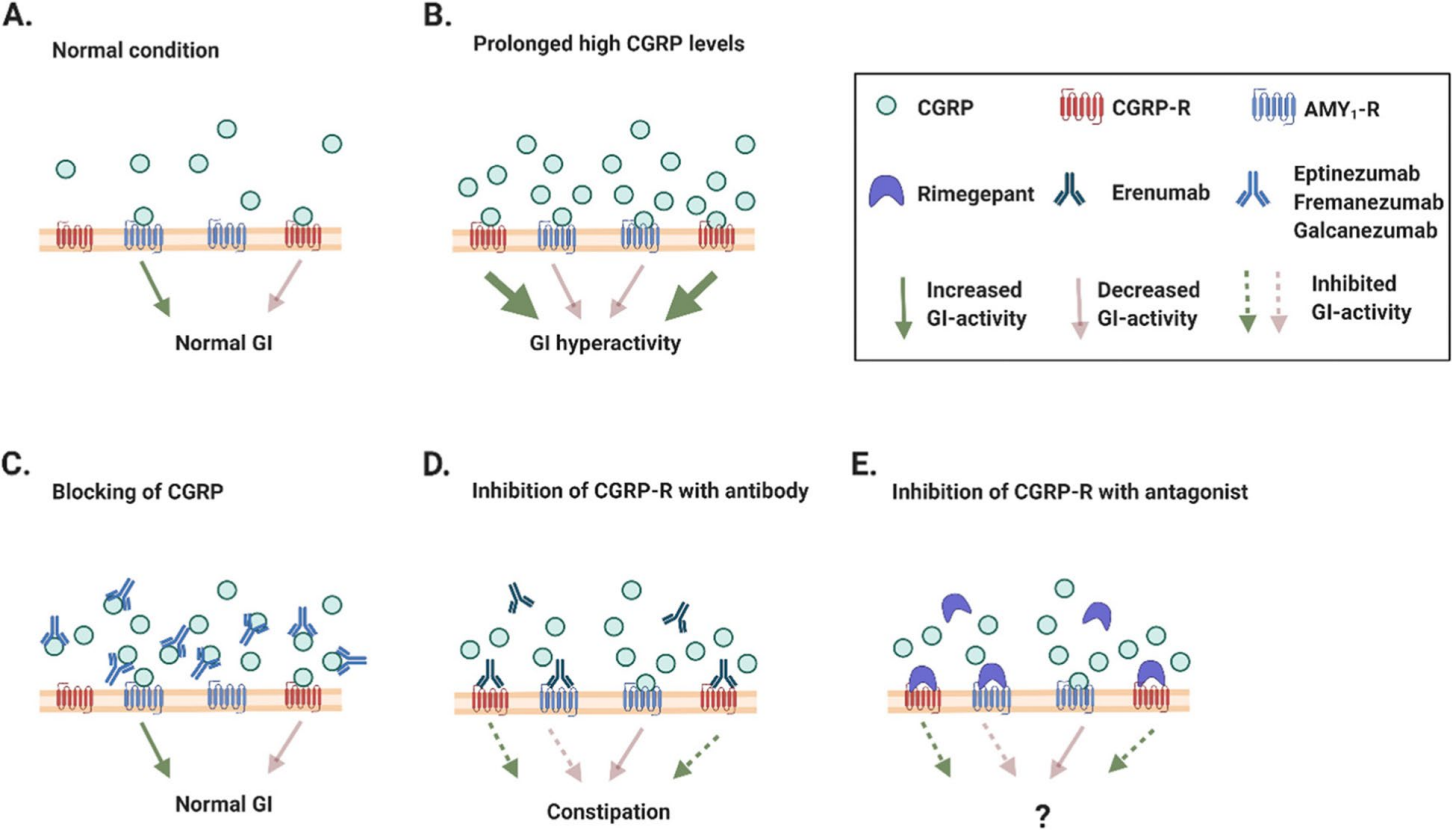
Schematic overview of the suggested role of CGRP and its antagonists in the gastrointestinal (GI) system. **A** Shows the normal situation with a low amount of free CGRP to stimulate the receptors **B** In a situation with high CGRP levels as described in [38] the CGRP molecules will bind to both CGRP-R and AMY_1_-R. The higher affinity of CGRP to the CGRP-R leads to GI hyperactivity as observed during infusion of CGRP for 2 hrs [38]. In **C** inhibition of the CGRP molecules with monoclonal antibodies (e.g. Eptinezumab, Fremanezumab or Galcanezumab) is shown. Less free CGRP leads to a lower binding rate of CGRP to CGRP-R and AMY_1_-R. The balance between signaling from CGRP-R and AMY_1_-R is intact and neither GI-hyperactivity nor constipation is found. **D** Treatment of patients with erenumab a monoclonal antibody directed towards the CGRP-R, prevents binding of CGRP to the CGRP-R. Erenumab can to some extent also block the AMY_1_-R. However, some CGRP molecules still binds to the free AMY_1_-R. This situation causes an imbalance of CGRP signaling in the GI system with a larger inhibition of CGRP’s stimulating effect on CGRP-R than CGRP’s antagonistic effect on AMY_1_-R. It should be noted that some of the AMY_1_-R mediated effects on the GI-system can be secondary via receptors in area postrema. The overall effect is constipation, which has been reported during treatment with erenumab [30, 31]. **E** Future Real-World studies will reveal to what extent constipation occurs after treatment with small molecule antagonists such as Rimegepant. According to the results of the present study a similar pattern as described in **C** seems likely

A 2 hour infusion of CGRP to healthy volunteers caused hyperactivity of the GI tract including diarrhea [38]. The steady state concentration of CGRP was reached after 60 min Simultaneous with the occurrence of GI adverse effects [38, 39]. In the present study, CGRP was 14 times more potent on the CGRP-R than on the AMY_1_-R. This plus unknown in vivo density and sensitivity of the receptors may explain the increased GI activity. Furthermore, we found Erenumab to be 32 times more potent on the CGRP-R than on the AMY_1_-R. Thus, the inhibition of CGRP evoked intestinal motility by erenumab might be stronger at the CGRP-R than at the AMY_1_-R which has a slowing effect on the GI-system [3]. In contrast, treatment with monoclonal antibodies against the CGRP molecule inhibits the effect of CGRP equally on the two receptors and therefore does not disturb GI function to the same degree (Fig. 7). Rimegepant is ~ 3 times more potent than erenumab on both receptors, and 25 times more potent on the CGRP-R than on the AMY_1_-R. Thus, according to our hypothesis our results suggest that prophylactic use of rimegepant may induce constipation. This was reported for another small molecule CGRP-R antagonist atogepant [40]. But, so far one study published on rimegepant for prophylactic use did not describe constipation as a side effect [40]. The reason for this could be explained by certain features of rimegepant as compared to erenumab such as higher dosing, a difference in pharmacokinetic properties, a smaller difference in affinity and a slightly higher potency on both receptors. However, rimegepant has been on the market for only a short time and future Real-World studies will reveal to what extent constipation occurs with this drug. Furthermore, we look forward to the results from future studies characterizing the effect of CGRP on AMY_1_-R in the gastro-intestinal tract contradicting or supporting our hypothesis.

## Conclusion

CGRP is 15-times more potent on CGRP-R than on AMY_1_-R expressed in *Xenopus laevis* oocytes. Amylin activates AMY_1_-R but not CGRP-R. The monoclonal antibody erenumab that is directed towards the CGRP-R and the CGRP-R antagonist rimegepant are both potent antagonists of CGRP-R and AMY_1_-R with 35- and 25-times preference for the CGRP-R over the AMY_1_-R, respectively.

## Supplementary Information

**Additional file 1: Table S1.** Summary of studies investigating the antagonistic effect of erenumab on α-CGRP induced responses on CGRP-R and AMY_1_-R. If nothing else is mentioned in the response column, the agonist used is α-CGRP and the antagonist is erenumab. **Table S2.** Summary of studies investigating the antagonistic effect of rimegepant on α-CGRP induced responses on CGRP-R and AMY_1_-R.

## Data Availability

The datasets used and analysed during the current study are available from the corresponding author on reasonable request.

## Abbraviations

AMY1-R: Amylin 1 receptor
cAMP: Cyclic adenosine monophosphate
CGRP-R: Calcitonin gene-related peptide receptor
CLR: Calcitonin receptor-like receptor
CTR: Calcitonin receptor
GI: Gastro-intestinal
CGRP: Calcitonin gene-related peptide
IC_50_: The concentration of a drug that gives half-maximal inhibition of a response
MCF-7: Michigan Cancer Foundation-7
RAMP1: Receptor activity-modifying protein 1
RAMP2: Receptor activity-modifying protein 2
RAMP3: Receptor activity-modifying protein 3
pIC_50_: The negative log of the IC50 value when converted to molar
TPM: Transcripts per million
