# Supplementary material for: Characterization of erenumab and rimegepant on calcitonin gene-related peptide induced responses in Xenopus Laevis oocytes expressing the calcitonin gene-related peptide receptor and the amylin-1 receptor
Source: J Headache Pain. 2022 May 26;23(1):59. doi: 10.1186/s10194-022-01425-9 (PMC9134599; doi:10.1186/s10194-022-01425-9)
Supplement: Supplementary file 1 — Additional file 1: Table S1. Summary of studies investigating the antagonistic effect of erenumab on α-CGRP induced responses on CGRP-R and AMY1-R. If nothing else is mentioned in the response column, the agonist used is α-CGRP and the antagonist is erenumab. Table S2. Summary of studies investigating the antagonistic effect of rimegepant on α-CGRP induced responses on CGRP-R and AMY1-R. [file 10194_2022_1425_MOESM1_ESM.pdf]

Supplementary material

Tabel S1: Summary of studies investigating the antagonistic effect of erenumab on α-CGRP induced responses on CGRP-R and AMY_1-R.

Cell type	Receptor	Response	$pIC_{50}^{(I)}$, $pEC_{50}^{(E)}$ or $pA_2^{(A)}$	CGRP-R /AMY_1-R	Reference
**MCF-7**	AMY_1-R	cAMP (calcitonin)	6.68 $^{(I)}$	-	[13]
**SK-N-MC**	CGRP-R	cAMP	8.64$^{(I)}$	-	[13]
	CGRP-R	cAMP	7.13$^{(I)}$	-	[14]
**HEK 293**	CGRP-R<br>AMY_1-R	Flow cytometry binding	8.64$^{(E)}$<br>7.38$^{(E)}$	18	[14]
**Oocytes**	CGRP-R + CFTR	Chloride current (α-$CGRP_{8-37}$)	8.4 $^{(A)}$	-	[9]
	CGRP-R<br>AMY_1-R	Chloride current	10.86$^{(I)}$<br>9.35$^{(I)}$	32	La Cour et al. Current study

If nothing else is mentioned in the response column, the agonist used is α-CGRP and the antagonist is erenumab.

Tabel S2: *Summary of studies investigating the antagonistic effect of rimegepant on* α-CGRP *induced responses on CGRP-R and AMY_1-R.*

Cell type	Receptor	Response	pIC_{50}	Ratio CGRP-R /AMY_1-R	References
**Cos7**	CGRP<br>AMY_1-R	cAMP	8.00<br>6.77	17	[15]
**Oocytes**	CGRP-R<br>AMY_1-R	Chloride current	11.30<br>9.91	25	La Cour et al. Current study
